# Work-Fitness Evaluation for Shift Work Disorder

**DOI:** 10.3390/ijerph18031294

**Published:** 2021-02-01

**Authors:** Tae-Won Jang

**Affiliations:** Department of Occupational and Environmental Medicine, Hanyang University, Seoul 04763, Korea; om1024@hanyang.ac.kr; Tel.: +82-31-560-2958

**Keywords:** shift work, sleep work disorder, sleep disturbances, circadian rhythm, circadian disruption, sleep diary, actigraphy, work-fitness

## Abstract

Shift work disorder (SWD), which is characterized by insomnia and excessive sleepiness related with shift work, is one of the most common health problems in shift workers. Shift work disorder causes insomnia, fatigue, worse work performance, an increased likelihood of accidents, and a poor quality of life. In addition, SWD is associated with decreased productivity and increased economic costs. The correct management of SWD is important to prevent sleep disturbances and maintain work performance in shift workers. To diagnose and evaluate SWD, it is necessary to take detailed medical histories, assess the severity of sleep disturbances, and evaluate shift workers’ sleep using a sleep diary and actigraphy. The work-fitness evaluation should include recommendations on how shift workers can reduce their sleep disturbances and increase work performance, as well as the assessment of work performance. This paper reviews previous research on the evaluation, diagnosis, and management of SWD and summarizes the work-fitness evaluation of SWD.

## 1. Introduction

In the 24/7 society in which we live, many people work day and night, be it for public safety and health services or for economic reasons. When an individual works shifts, the internal clock might not match the external time, which can disrupt the circadian rhythm [1]. This circadian rhythm desynchronization can have various health consequences, including sleep disturbances, cardiovascular disease, gastrointestinal disorders, mental problems, and breast cancer [2,3,4,5,6].

Shift work disorder (SWD) is one of the most common health problems in shift workers [7]. Shift work disorder causes insomnia, fatigue, a lower job performance, and an increased likelihood of accidents, all of which diminish quality of life [8,9]. In addition, SWD is associated with productivity loss and increased economic costs [10]. Recently, various interventions to manage SWD have been proposed. Organizational interventions include adjustment of the shift work schedule and working hours, taking naps and rest breaks during night shifts, and offering commute vehicles [11,12]. The workplace can also provide health promotion programs for SWD such as education about how to attain good sleep quality and meditation programs [13]. Clinicians have also explored various potential treatment options for SWD. Non-pharmacological interventions include cognitive behavioral therapy for insomnia, scheduled sleep, controlling the sleep environment and sleep hygiene, napping before night shifts, circadian realignment, and light therapy. Pharmacological interventions include hypnotics, anti-depressants, and melatonin [14,15,16,17,18,19,20].

Both organizational and individual interventions are important for the management of SWD. From the standpoint of occupational medicine, the proper placement of workers and work-fitness evaluations are crucial for SWD management. There have been many studies on the treatment and management of SWD, but few studies have been conducted on the work-fitness evaluation of SWD. This paper reviews research on the evaluation, diagnosis, and management of SWD and summarizes the work-fitness evaluation of SWD.

## 2. Circadian Rhythm and Shift Work

The circadian rhythm is a physiological cycle that repeats approximately every 24 h. Many processes, including the sleep–wake cycle, hormonal changes, body temperature, and blood pressure, follow the circadian rhythm [21]. The suprachiasmatic nuclei (SCN) in the hypothalamus, which is called the internal or circadian clock, regulates the human circadian rhythm [22]. Circadian rhythm is affected by various environmental cues including light, temperature, work and rest, and eating. Among these cues, light has the greatest influence on the circadian rhythm [23]. During the day, the SCN detects light that enters the eyes through the retinohypothalamic tract and stimulates the release of melatonin from the pineal gland; when it is dark, for example at night, the SCN inhibits pineal gland activity, which stops melatonin secretion [24]. Melatonin promotes sleep through a decrease in core body temperature and an increase in peripheral heat loss [25].

When the timing of the external environmental does not match the human internal clock, the circadian rhythm begins to adjust, such that the internal clock meets the environmental time. The mismatch between the human internal clock and the environmental time is called external desynchronization, and the adjustment of the internal clock to meet the environmental time is called re-entrainment [26].

During re-entrainment, human circadian rhythms of various physiological processes change and adjust to meet the environmental time, thus deviating from the internal clock. However, the circadian rhythms of each physiological process do not coordinate at the same rate; some rhythms with large exogenous components such as the sleep–wake cycle and eating adjust rapidly, whereas rhythms with large exogenous components, such as hormone secretion and body temperature, adjust more slowly [27].

It takes considerable time for all rhythms to become completely attuned to the environmental time; until that point, the re-entrainment of the circadian rhythm is not complete, and the circadian rhythm system does not work efficiently. During this period, the individual processes governed by the circadian rhythm do not match each other; this state is called internal desynchronization, or circadian disruption [28]. Internal desynchronization is the most influential cause of shift work intolerance [29].

Individuals who travel long distances on trans-meridian flights can feel uncomfortable while adjusting to new time zones, which is known as jet lag disorder. When re-entrainment is complete, the symptoms of jet lag disorder disappear. Shift workers’ internal clock is often different from the environmental time, and they may therefore experience symptoms that are similar to those of jet lag disorder. However, unlike long-distance travelers, shift workers must adjust to a new time zone whenever they start a new shift, which disrupts their circadian rhythm. When a worker starts working at night, the circadian rhythm shifts forward. Conversely, when a worker finishes night work and starts day work, the circadian rhythm shifts backward. The entrainment of internal clock is a slow process, and many days are needed to adjust to an environmental time zone [30]. Many shift workers change their work schedule before the internal clock completes re-entrainment, and as a result, the internal clock starts re-entrainment to a different time zone. During this re-entrainment, shift workers experience various symptoms related to circadian rhythm misalignment.

## 3. Definition and Diagnosis of SWD

### 3.1. Definition

The *International Classification of Sleep Disorders*, Third Edition (ISCD-3), classifies SWD as one of the circadian rhythm sleep–wake disorders [31]. The definition of SWD is as follows:

(1) There is an insomnia or/and an excessive sleepiness with a reduction of total sleep time, all combined with an overlap of work period occurring during the habitual sleep time;
(2) The presence of these symptoms has lasted for at least 3 months and are associated with the shift work schedules;
(3) Sleep log or actigraphy monitoring (with sleep diaries) demonstrate for more than 14 days (work and free days included) circadian and sleep-time misalignment;
(4) Sleep disturbance is associated with impairment of social, occupational, and/or other waking functioning;
(5) These symptoms are not better explained by another sleep disorder, medical or neurologic disorder, mental disorder, medication use, poor sleep hygiene, or substance use disorder.

### 3.2. Diagnosis

The *Diagnostic and Statistical Manual for Mental Disorders*, Fifth Edition (DSM-5), describes the following diagnostic criteria for circadian rhythm sleep–wake disorder and SWD [32]:

General criteria for circadian rhythm sleep–wake disorder:

A. A persistent or recurrent pattern of sleep disruption that is primarily due to the fact of an alteration of the circadian system or to a misalignment between the endogenous circadian rhythm and the sleep–wake schedule required by an individual’s physical environment or social or professional schedule;
B. The sleep disruption leads to excessive sleepiness or insomnia, or both;
C. The sleep disturbance causes clinically significant distress or impairment in social, occupational, and other important areas of functioning.

Specific criteria for shift work type: 307.45 (G47.26)

Insomnia during the major sleep period and/or excessive sleepiness (including inadvertent sleep) during the major awake period associated with a shift work schedule (i.e., requiring unconventional work hours).

Careful examination of occupational and medical history is most important for a diagnosis of SWD. Sleepiness at work and sleep disturbances at home are prominent features, and so the presence of both symptoms is necessary for a diagnosis of SWD [32]. Sleep disturbances may vary according to the change of shift and usually persist on rest days as well as working days. Fatigue and sleepiness may decrease an individual’s job performance and induce accidents, both on duty and on the drive home [33]. Individuals with SWD may engage in undesirable health behaviors such as smoking, heavy alcohol drinking, and lack of exercise [34]. Shift work disorder is known to be associated with mental disorders such as depression and anxiety, and the evaluation of mental health should therefore be considered for the differential diagnosis and management of SWD [5].

Polysomnography is a good method by which to diagnose sleep disorders [35]. For the diagnosis of SWD, polysomnography can help to detect comorbidities such as sleep apnea syndrome and restless lag syndrome. However, this method has limitations for the evaluation of SWD, because sleep disturbances can vary according to an individual’s type of shift, and sleep in the laboratory may be different from sleep at the workplace or at home. Although the actigraphy does not provide as much information as polysomnography, it was validated for the assessment of sleep and useful for the diagnosis of SWD [36,37]. Actigraphy is useful to evaluate sleep disturbances of both working days and rest days. However, actigraphy is not essential for diagnosis of SWD, and detailed medical and occupational history is most important as described above.

## 4. Evaluation of SWD

### 4.1. Assessment of Sleep Disturbances

There are many instruments to assess sleep disturbances. Such measures include the Insomnia Severity Index, Mini Sleep Questionnaire, Epworth sleepiness scale, Functional Outcomes of Sleep Questionnaire, Pittsburgh Sleep Quality Index, Athena Sleep Questionnaire, Sleep Apnea Clinical Score, Berlin Questionnaire, Shift Work Disorder Screening Questionnaire, and Bergen Shift Work Sleep Questionnaire [38,39,40,41,42,43,44,45,46,47] (Table 1). Among these, the SWDSQ and BSWSQ were specifically developed to evaluate SWD in shift workers.

The SWDSQ was developed for use in primary care settings for the diagnosis of SWD. The items assess the difficulty of falling asleep due to the fact of waking up too early, sense of well-being, dozing off at work, and dozing off while driving in the past month. The SWDSQ consists of four items, which makes it easy to complete, and is suitable for the screening of SWD [46].

The BSWSQ was developed to assess sleep disturbances, fatigue, and sleepiness in relation to different work shifts. The items assess sleep latency, wake after sleep onset, sleep quality, fatigue on duty and rest breaks, and fatigue on rest days in the past 3 months. Responders respond to each item with the frequency of each symptom during the day, evening, night shifts, and rest days. The BSWSQ can evaluate the frequency of various sleep disturbances according to the type of shift, which is suitable for the evaluation of SWD severity [47].

### 4.2. Sleep Diary

Keeping a sleep diary can help to identify sleep patterns of shift workers according to the changes in their work shift [7]. Sleep diaries should include information about the time to bed, time spent falling asleep, wake-up time, how long it takes to get out of bed, and wakefulness during the sleep period. It should also include daily information about the start and end time of the shift or whether it was a rest day. A sleep diary should be completed for at least 2 weeks, and if the cycle of shift work exceeds 2 weeks, it should be completed for the period required to cover all shifts.

Information that can be obtained from a sleep diary includes sleep latency, sleep duration, sleep quality, and duration and frequency of awake during sleep. These daily parameters should be recorded longitudinally, which can be helpful in identifying sleep patterns of shift workers on both shift and rest days. Since the sleep diary is recorded by the patient, the information may have some limitations such as incomplete record and inaccuracy due to the fact of recall and confirmation bias.

### 4.3. Actigraphy

**Figure 1:**
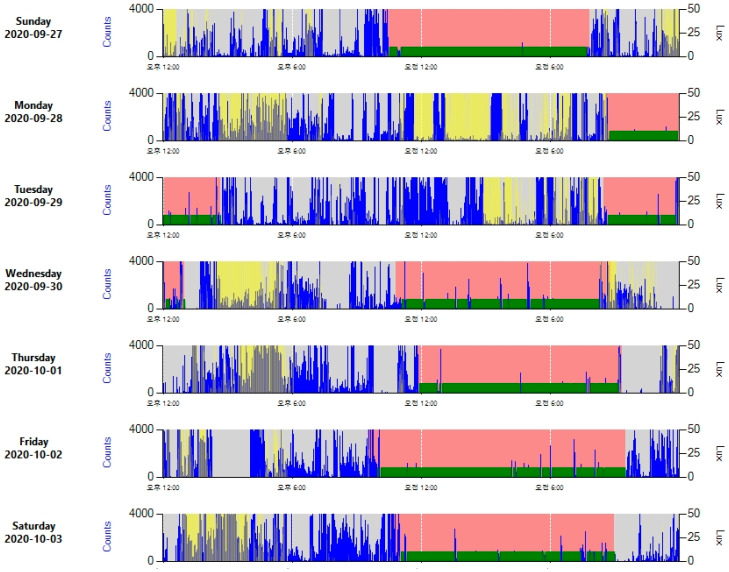
Sample of actigraphy, measured with ActiGraph (WGT3X-BT; ActiGraph, 49 E, Chase St. Pensacola, FL, USA). Blue shows body movements, red and green color indicate the sleep period, and yellow indicates the detection of light.

Actigraph is a device that detects body movements and is worn on the wrist of the non-dominant hand. Actigraphy refers to measuring and recording body movements for days or weeks using an actigraph (Figure 1). Actigraphy is a useful objective method to measure sleep patterns of shift workers, and actigraphs can be easily worn by shift workers while sleeping at work or at home [36]. Actigraphy should be measured for at least 2 weeks or for a sufficient period to cover all types of shift work, much like a sleep diary.

Actigraphy should be measured along with a sleep diary. Sleep duration (the time period from when the subject went to bed until when they got out of bed) cannot be determined from the actigraphy alone. The computer program for sleep analysis requires not only the actigraph measurements but also information from the sleep diary.

Parameters derived from actigraphy are described in Table 2 [48]. Sleep latency (SL), also called sleep onset latency, is the time from when an individual attempts to sleep and actually falls asleep. The SL of normal adults is approximately 10–20 min, an SL of 0–5 min indicates severe sleep deprivation, and the American Academy of Sleep Medicine defines an SL < 8 min as sleepiness [49]. If the SL is longer than 20–30 min, this indicates insomnia, with difficulty initiating sleep [32].

Time in bed (TIB) is the duration of time from going to bed and getting out of bed. Total sleep time (TST) is the duration of time spent in actual sleep. Sleep efficiency (SE) is the percentage of time spent asleep while an individual is in bed. Wake after sleep onset (WASO) is the amount of time spent awake during the sleeping period after sleep onset. In normal adults, the WASO is <10% of the sleep period (TIB) and increases with age [50]. According to a study by Mitterling et al. [50], SE and WASO% (the percentage of time spent WASO of the total TIB) were 87.1% and 8.5% in people 31–40 years of age, whereas they were 79.7% and 15.2% in those >60 years of age, respectively.

Total sleep time is calculated as TIB minus SE and WASO. Wake after sleep onset comprises approximately 8–15% of TIB in normal adults, so SE should be >85%. A low SE indicates poor sleep quality, whereby a lower SE indicates worse sleep quality. A low SE may result from both an increased SL and an increased WASO. Sleep onset insomnia refers to difficulty falling asleep when individuals attempt to initiate sleep [51]. Sleep onset insomnia can be diagnosed when an individual cannot fall asleep after spending 20–30 min attempting to sleep. In patients with sleep onset insomnia, the SL increases and the SE decreases.

Wake after sleep onset is the duration spent awake after sleep onset, which indicates sleep fragmentation. Thus, a high WASO indicates poor sleep quality, whereby a higher WASO indicates a worse sleep quality. Sleep maintenance insomnia is the difficulty to stay asleep during sleep period Individuals with sleep maintenance insomnia experience waking up during the sleep period and struggling to get back to sleep for at least 20–30 min. In patients with sleep maintenance insomnia, WASO increases and SE decreases.

### 4.4. Assessment of Circadian Rhythm

Assessment of the circadian rhythm is not essential for a diagnosis of SWD. However, it is helpful to identify how the circadian rhythm changes in shift workers according to different types of shift work. There are several methods to assess circadian rhythm, such as evaluation of the sleep–wake (rest–activity) cycle measurement of hormones such as cortisol and melatonin, and measurement of body temperature [52,53,54,55].

Cortisol is an important hormone that regulates cardiovascular, metabolic, immunologic, and homeostatic functions [56]. Serum cortisol rises rapidly during early morning and peaks 30–45 min after awakening, which is called the cortisol awakening response (CAR) [57]. Cortisol then decreases in the evening and during the early phase of sleep [58]. Circadian rhythm can be assessed by measuring the CAR.

Melatonin is associated with the circadian rhythm and circadian clock in humans and plays an important role in sleep regulation in humans [59,60]. In general, the increase in sleep propensity occurs 2 h after the onset of melatonin secretion in humans [61]. For this reason, individuals with SWD are advised to take melatonin 2 h before sleep. The diurnal variation of melatonin is closely associated with the circadian rhythm; melatonin is released during the night, so serum melatonin is high at night and low during the day [62]. Circadian rhythm can be assessed by measuring the dim light melatonin onset (DLMO), which means the onset of melatonin secretion under dim light condition [63].

To evaluate the circadian rhythm, it should be decided which specimen (saliva, blood, or urine) will be measured, over how many days the assessment will take place, and how many times a day measurements will be taken. The circadian rhythm of a shift worker changes according to the work schedule, and the CAR and DLMP also change. Therefore, the higher the measurement frequency, the more accurately the circadian rhythm can be evaluated. However, it is impractical to collect specimens from a subject multiple times per day, and even more so over multiple days. In addition, cortisol and melatonin measurement is expensive [52]. For this reason, the measurement of cortisol or melatonin is not an optimal method by which to assess the circadian rhythms of shift workers.

The diurnal variation of core body temperature is different from distal body temperature. Body temperature variation is determined by heat production and loss of the human body. At night, heat radiation through the skin increases, so core body temperature decreases and distal body temperature increases. Conversely, during the day, as heat radiation through the skin decreases, the core body temperature increases and distal body temperature decreases [64]. As a result, the core body temperature is lowest and distal body temperature is highest at dawn; whereas the core body temperature is highest and distal body temperature is lowest in the late afternoon [65].

Body temperature is one of best markers for the human circadian rhythm and has been used to evaluate the circadian rhythm in humans [52]. Body temperature measurement is a non-invasive method; can be measured almost continuously, even over multiple days; and is cost-effective. Therefore, body temperature measurement is a good method to evaluate changes in shift workers’ circadian rhythm according to shift changes [66,67,68].

### 4.5. Others

When evaluating SWD, it is helpful to assess comorbidities such as fatigue, sleep apnea, restless leg syndrome, mental conditions, cardiovascular diseases, and gastrointestinal disorders. Tools to evaluate sleep problems and mental conditions include the Fatigue Severity Scale, Sleep Apnea Clinical Score, Berlin Questionnaire, Restless Legs Syndrome Rating Scale, Patient Health Questionnaire-9, Beck Depression Inventory, and Generalized Anxiety Disorder-7 [44,45,69,70,71,72,73].

## 5. Fitness for Work

Work-fitness evaluation is the assessment of an individuals’ ability to work without risk to their own or others’ health and safety [74]. Typically, the outcome of a work-fitness evaluation can be (1) fit for work, (2) fit for work with conditions or restrictions, or (3) not fit for work, temporarily or permanently [75]. The main purpose of this assessment was to identify whether individuals were not fit for work due to the risk of adverse health effects and to assign them appropriate work. Far more workers are fit for their work than those who are not. Therefore, the work-fitness evaluation should not only be used to identify workers who are not fit for work, but also to recommend workers without adverse health effects who can perform their work.

Work-fitness evaluation of SWD aims to find workers who have difficulties adapting to shift work and to help shift workers to alleviate health problems associated with SWD. For this, the physician performing work-fitness evaluation should be aware of the factors to consider in deciding the recommendations on whether to allow the workers to continue shift work or not. In addition, it is also important to recommend appropriate methods to alleviate the symptoms of SWD to shift workers.

### 5.1. Evaluation for Work-Fitness

Shift work disorder is common in shift workers, but not all shift workers experience SWD. Some workers cope well with shift work, while others cannot adapt to shift work and can experience moderate to severe sleep disturbances and cardiovascular and gastrointestinal conditions. Shift maladaptation syndrome refers to the inability to adapt to shift work due to the fact of sleep disturbances, family and social disharmony, health conditions including gastrointestinal and cardiovascular disease, and poor work performance [76]. Intolerance to shift work has several characteristics as follows [77]:

(1) Sleep disturbances including poor sleep quality, difficulties falling asleep, and frequent awakenings;
(2) Fatigue, even after sleep, weekends, and vacations;
(3) Changes in behavior, including unusual irritability, malaise, and a feeling of inadequacy;
(4) Gastrointestinal troubles including dyspepsia, epigastric pain, and gastric ulcers;
(5) Regular use of sleeping pills such as benzodiazepines, phenothiazines, and tranquilizers, which cannot resolve sleep disturbances.

The decision of work fitness should be made by considering a worker’s sleep disturbances, fatigue, mental conditions, and comorbidities such as gastrointestinal and cardiovascular conditions. Sleep disturbances, fatigue, and mental conditions can be evaluated by history taking and the assessment tools described above. Sleep disturbances and fatigue are usually evaluated using questionnaire tools which are tools to evaluate subjective symptoms of the patients, so objective methods may be needed to confirm the severity of SWD. The actigraphy are good objective methods to assess sleep disturbances of shift workers on both shift and rest days [36]. Another objective sign of shift work intolerance is the regular use of sleeping pills [77]. Thus, reviewing medical records to check for sleep disturbances of shift workers and determining whether they have regularly taken sleeping pills can help to identify shift work intolerance. Shift workers who experience moderate to severe sleep disturbances, even after taking medications for sleep disturbances, may not be fit for shift work.

### 5.2. Recommendations for SWD

Some workers can perform shift work without any discomfort, and most workers are forced to work shifts, even if they have SWD. In other words, the most frequent outcome of the work-fitness evaluation for SWD is “fit for work with conditions or restrictions”. For this reason, it is important to recommend appropriate conditions to workers with SWD to relieve sleep disturbances and improve work performance.

The purpose of making recommendations on how to deal with SWD is to improve the fitness of shift workers and help them to work without adverse health hazards during shift work. The recommendations may be in the form of an organizational intervention, such as adjusting the shift work schedule and providing enough rest breaks, or an individual intervention such as sleep hygiene and taking medications.

The National Institute for Occupational Safety and Health and Health and Safety Executive have published a guide for shift workers [12,78]. Some important organizational interventions in these guidelines are as follows:

(1) Permanent shift should be avoided;
(2) Concerning rotating shift work, a fast rotation every 2–3 days or a slow rotation at least every 3–4 weeks is recommended, and weekly rotation is not recommended. In addition, forward rotation is better than backward rotation;
(3) Long working hours should be avoided: 12 h shifts should be limited to 2–3 successive nights, and shifts longer than 12 h should be avoided;
(4) There should be at least 11 h between each shift to allow workers enough time to sleep between shifts;
(5) Physically demanding, monotonous, or dangerous work should not be assigned to night work;
(6) Regular free weekends should be provided to workers for their family and social life;
(7) The shift work schedule should be designed as regularly as possible, and shift workers should be informed of their work schedules in advance. If possible, shift workers should have the right to adjust their schedule.

In addition to the above, it can be considered to arrange workers to work schedule in consideration of their chronotype [79]. For example, workers of morningness type can be assigned to morning or day work, and workers of eveningness type can be assigned to night work.

Organizational intervention cannot be performed by individual workers themselves, who must therefore discuss with other staff or the manager in the workplace when recommending organizational interventions. Occupational physicians should advise the manager or staff of the workplace on the strategy for the management of SWD.

Individual interventions include making a management plan to resolve sleep disturbance and improving work performance for shift workers. The management for SWD includes sleep strategies for shift workers, sleep hygiene, and medication.

Given that sleep deprivation is common in shift workers, naps may be an effective strategy for supplementing sleep. There have been many studies on napping during night shifts. Although some studies have shown that naps are not effective in supplementing sleep, most studies have reported that napping during night shifts is associated with a decrease in sleep disturbances and fatigue, increased alertness, and better job performance [80,81,82,83,84]. The appropriate length of naps has been inconsistently reported by researchers, and short naps of 20–30 min may be advisable [80,85,86]. Naps > 20–30 min can lead to a deeper sleep (stages 3 and 4), which may cause sleep inertia after napping [87]. On the other hand, it takes about 90 min to complete a sleep cycle (stages 1–4 and REM sleep), so long naps of 90 min are also effective [88]. Therefore, short naps of 20–30 min or a long nap of 90 min seem to be most appropriate for shift workers, if the workplace can provide an appropriate environment in which to nap during night shifts. In addition, some time should be provided to fully recover from napping before returning to work.

If workers are not allowed to nap during night shifts, naps before starting the night shift are a good option. Naps of 1–4 h before night shifts, which have been called “prophylactic naps”, have been reported to improve performance and decrease sleepiness [89,90,91,92,93]. Therefore, workers can be recommended taking a nap of 90 min to 2 h before going to work at night, which could help to reduce sleepiness and enhance performance during night shifts.

Bright light therapy and melatonin administration is another option for shift workers with SWD. In general, exposure to light at late evening induces a circadian rhythm delay, and exposure to light in the early morning induces a circadian rhythm advancement [54]. Exposure to light before night shifts in shift workers induces a delay in their circadian rhythm and suppresses the secretion of melatonin, which can maintain alertness and performance during night shifts. On the other hand, exposure to light in the morning after night shifts prevents the circadian phase delay and induces circadian disruption [94,95]; thus, shift workers should block lights with sunglasses or blue-light blocking glasses after night shifts. However, wearing sunglasses while driving after night shift may increase the risk of accidents, so it is recommended not to drive after night shift. Light therapy has different effects depending on the intensity of light, exposure duration, and the timing of exposure, and the circadian rhythm may become even more disrupted when light therapy is applied incorrectly, so care should be taken.

Melatonin can also advance or delay the human circadian rhythm: melatonin administration at night induces circadian phase advancement and melatonin administration in the early morning induces circadian phase delay [96]. For shift workers, administration in the morning after a night shift can help them to adapt to the night shift schedule and increase sleep quality [97,98]. The hypnotic effect of melatonin appears 30 to 120 min after administration, so shift workers should take melatonin 1 to 2 h before sleep [99]. Melatonin administration requires a detailed explanation on the proper dosage and timing, and possible side effects such as nightmares, so the recommendation should be done with the consultant to medical doctors.

Shift work disorder with comorbidities, such as depression, anxiety, or sleep apnea, requires treatment of the comorbidity for the improvement of sleep disturbances. If necessary, the recommendation should be to consult with psychiatrists for the treatment of comorbidities.

## 6. Conclusions

The proper management of SWD can help to prevent sleep disturbances and maintain work performances for shift workers. The management should include proper placement of workers with shift work intolerance and management of sleep disturbances in the workers with SWD. For this, work-fitness evaluation should include both an assessment of work-fitness and the management plan for the workers with SWD. This paper may help the health managers in the workplace and the physicians of occupational medicine to manage and prevent SWD in shift workers.

## Figures and Tables

**Table 1:** Questionnaires for the evaluation of sleep disorders.

Instruments	Items	Evaluation
Insomnia Severity Index (ISI)	7	Insomnia
Mini Sleep Questionnaire (MSQ)	10	Insomnia and hypersomnia
Epworth Sleepiness Scale (ESS)	8	Daytime sleepiness
Functional Outcomes of Sleep Questionnaire (FOSQ)	10	Sleepiness on daily life
Pittsburgh Sleep Quality Index (PSQI)	9	Sleep quality
Athena Sleep Questionnaire (ASQ)	8	Sleep quality
Sleep Apnea Clinical Score (SACS)	4	Sleep apnea
Berlin Questionnaire (BQ)	10	Sleep apnea
Shift Work Disorder Screening Questionnaire (SWDSQ)	4	Shift work disorder (SWD) screening
Bergen Shift Work Sleep Questionnaire (BSWSQ)	7	SWD symptom severity

**Table 2:** Parameters of sleep actigraphy.

Parameters	Definition
Sleep latency (SL, minutes)	The duration between attempts to sleep and falling asleep
Time in bed (TIB, minutes)	The duration between time to bed and getting out of bed
Total sleep time (TST, minutes)	The total amount of time spent in sleep
Sleep efficiency (SE %)	Percentage of time spent asleep while in bed
Wake after sleep onset (WASO, minutes)	Period of awakening occurring after sleep onset
Number of awakenings	Frequency of awakenings after sleep onset
Length of awakenings (minutes)	Average duration of awakenings after sleep onset
